# Does weed diversity mitigate yield losses?

**DOI:** 10.3389/fpls.2024.1395393

**Published:** 2024-07-12

**Authors:** Marie L. Zingsheim, Thomas F. Döring

**Affiliations:** Institute of Crop Science and Resource Conservation, Agroecology and Organic Farming Group, Bonn, Germany

**Keywords:** weed management, weed communities, biodiversity, weed evenness, competitive effects

## Abstract

While intensive control of weed populations plays a central role in current agriculture, numerous studies highlight the multifaceted contribution of weeds to the functionality and resilience of agroecosystems. Recent research indicates that increased evenness within weed communities may mitigate yield losses in contrast to communities characterized by lower diversity, since weed species that strongly affect crop yields, also dominate weed communities, with a concurrent reduction of evenness. If confirmed, this observation would suggest a paradigm shift in weed management towards promoting higher community diversity. To validate whether the evenness of weed communities is indeed linked to higher crop productivity, we conducted two field experiments: one analyzing the effects of a natural weed community in an intercrop of faba bean and oat, and the other analyzing the effects of artificially created weed communities, together with the individual sown weed species, in faba bean, oats and an intercrop of both crops. The evenness of the weed communities ranged from 0.2 to 0.9 in the natural weed community, from 0.2 to 0.7 in faba bean, from 0 to 0.8 in the intercrop and from 0.3 to 0.9 in oats. Neither the natural nor the artificial weed community showed significant effects of evenness on crop grain yield or crop biomass. The results of this study do not validate a positive relationship of crop productivity and weed evenness, possibly due to low weed pressure and the absence of competitive effects but suggest that also less diverse weed communities may be maintained without suffering yield losses. This is expected to have far reaching implications, since not only diverse weed communities, but also higher abundances of few weed species may contribute to ecosystem functions and may support faunal diversity associated with weeds.

## 1 Introduction

With the establishment of farmland, humans created agroecosystems that differ from natural ecosystems by high disturbance frequencies and high resource availabilities through tillage and fertilization (Wet and Harlan, 1975). The resulting niches of agroecosystems are occupied by a wide variety of weeds species (O’Brien and Laland, 2012). Weeds compete with crops for light, water and nutrients, and infestation of weeds has a global potential yield loss of 34% (for wheat, rice, maize, potatoes, soybeans, and cotton) (Oerke, 2006). Therefore, weeds are controlled by several direct actions such as mechanical and chemical intervention, or indirect control measures including use of diverse crop rotations and breeding highly competitive crops (Liebman and Dyck, 1993; Wolfe et al., 2008; Gianessi, 2013; Naruhn et al., 2021). To keep economic costs of management interventions low, agricultural systems have tended to develop towards monocultures on which herbicides and fertilizers can be applied quickly over large areas. While efficient weed management does have its justification for ensuring food production in sufficient quantity and quality, weeds are not only detrimental but provide resilience and functionality of agroecosystems (Gerowitt et al., 2003; Storkey and Neve, 2018; Ilic, 2023). The different plant parts of weeds form the food basis for herbivores (Gaba et al., 2019) and thus for higher trophic levels e.g. birds (Wilson et al., 1997; Siriwardena et al., 1998). In addition, weeds provide reproduction sites and shelter to associated fauna including pollinators and natural predators of crop pests (Rebek et al., 2006; Holzschuh et al., 2013) and contribute to the reduction of soil erosion (Mendez and Buschiazzo, 2015; Lenka et al., 2017). This long-term functionality of agroecosystems is at risk. The intensification of agricultural systems has been recognized as a significant factor contributing to the global decline in biodiversity (Hallmann et al., 2017; Wagner et al., 2021) as it largely destroyed the diverse supply of niches, has led to herbicide resistances and the emergence of few dominant and highly competitive weed species (Foley et al., 2011; Storkey and Neve, 2018). Thus, there is a need to take actions in agriculture, to secure the functionality of agro-ecosystems either within or outside fields (e.g. flower strips). The ongoing debate on land sparing vs. land sharing has highlighted the advantages and disadvantages of biodiversity-promoting actions within versus outside production fields. Both approaches do have their justification (Grass et al., 2021). One land-sharing action that has the potential to contribute securing functionality is the development of weed management strategies that consider both food supply and biodiversity conservation in the field. These two objectives are not necessarily incompatible and recent research has shown that not all weed species and communities are detrimental to crop production although these findings are context dependent and many species can be harmful under specific conditions (Boström et al., 2003; Esposito et al., 2023). Nevertheless, it is evident that diverse weed communities implicate a high diversity of traits, which limits the intensive niche overlap with crop plants compared to communities dominated by highly competitive species that are strongly adapted to a specific cropping system (Smith et al., 2010; Navas, 2012). In particular, Navas (2012) suggested that “high [trait] divergence inducing complementarity in resource use by weeds and crop across time or space, in relation to niche differentiation, should result in a reduced impact of weeds on crops”. In accordance with this, Adeux et al. (2019) found that with increasing evenness within a weed community, weed biomass decreased by 83% and crop productivity increased by 23%. Similarly, already (Cierjacks et al., 2016) found positive correlations between weed evenness and banana and coconut yields. These findings are thus also in line with theoretical expectations that weed traits conferring high competitiveness against crops, especially under nutrient-rich conditions, would also tend to suppress other weed species, thereby reducing community evenness. In practical terms, these findings might offer an in-field trade-off reduction by managing weeds towards a diverse weed community without suffering yield losses; we therefore see these results as potentially promising for future research on integrated weed management. On the other hand, in a comprehensive study on the effects of weeds on multifunctionality in agroecosystems, Gaba et al. (2020) reported that “weed diversity had no significant effects on [ … ] oilseed rape fruiting success”, as a measure of crop productivity. Further, correlations between weed species richness and crop yield were found to be non-significant so far (Cierjacks et al., 2016; Adeux et al., 2019; Gaba et al., 2020; Stefan et al., 2021). Finally, only a few studies have so far tested relationships between weed diversity and crop yield, and, despite some significant results, these are characterized by large variance (Cierjacks et al., 2016; Adeux et al., 2019). Thus, the conclusions of these studies remain somewhat uncertain so far. Therefore, further research is needed to consolidate the picture of how weed diversity and crop productivity are related. In particular, without a comprehensive research base, the willingness of farmers to maintain weeds on their fields will remain low due to concerns about yield losses caused by weed infestation.

The aim of this study was to validate the relationship between diversity of the weed community, especially the evenness, and crop productivity by conducting two field experiments. In one experiment the natural appearing weed community was investigated, whereas in another experiment, an artificial weed community was established and studied. This enabled the measurement of both species-specific effects and the effects of weed communities varying in evenness on the crops’ productivity.

## 2 Materials and methods

To investigate the effects of weed evenness on crop yield, both natural and artificial weed communities were investigated in separate, complementary field experiments at different locations. The experiment in a natural weed community enables the investigation of effects between weed species and crops as they occur naturally. However, the natural heterogeneous distribution of weeds restricts the separation of the evenness effects from species-composition effects as these compositions vary among the investigated plots. The second experiment with an artificial community enabled this separation as species-composition remains (almost) constant and only the evenness differs between plots. The range of contexts in which the experiments were conducted was increased by including two different experimental locations and three different cropping systems in this study.

### 2.1 Natural weed community

#### 2.1.1 Experimental field site

Both experiments were located in west Germany with a distance of approximately 50 km to each other. The investigation of the natural weed community was conducted on the research station for Organic Farming ‘Campus Wiesengut’ of the University of Bonn in Hennef, Germany. The local climatic conditions are characterized by a mean annual temperature of 10.3°C and a mean annual precipitation of 840 mm. The Wiesengut farm is located at 50°47.2’ N, 7°16.5’ E with an altitude of 65 m a.s.l. in the lowland of the river Sieg. The site is characterized by a ‘Fluvisol’ soil with a silty loam texture on gravel layers with soil depth of 0.6 to 2.0 m and fluctuating groundwater level. The particular field was chosen because its soil texture was known to be strongly heterogeneous, with the depth of the gravel layer varying greatly across the field. Previous field experiments have shown a spatial heterogeneity of the weed community composition as well (Zingsheim and Döring, 2024), which in turn was expected to form the basis of a high variation in evenness.

#### 2.1.2 Setup

The experiment was performed in a uniform regular grid (12 m distance between grid points), with 44 grid points, within an area of 72 m x 108 m in spring-sown intercrop of faba bean (cv. Fanfare) and oat (cv. Max). The previous crops sown at the site were winter wheat in 2018 and winter rye in 2019. The seedbed was prepared with a rotary harrow; no fertilization, irrigation or direct weed control was carried out.

#### 2.1.3 Data acquisition

The vegetation was surveyed on two dates (**Table 1**) at each grid point to record both early and later germinating species. At each grid point, sampling was performed on a plot size of 2 m x 2 m, so that the grid point was the plot center (Zingsheim and Döring, 2024). The frequency of present species is presented in the **Supplementary Material** (**Supplementary Figure A3**). Crop emergence (i.e. crop density) was counted in two rows for two meters and expressed as plants per square meter. The biomass of crops and weeds was measured on 0.25 m² at each grid point on June 9^th^, 2020, by cutting off plants just above the soil surface. Crops and weed plants were in flowering stage at this time. The plants were separated into faba bean, oat and weed, then fresh and dry mass of the plants were measured. For determining dry mass, the plants were oven-dried for 12 hours at 60°C and then for 12 hours at 105°C. Furthermore, the species-specific cover of weed plants was estimated on to dates (**Table 1**).

**Table 1:** Experimental details with sowing density, sowing and harvest date, and sampling dates.

	Natural community	Artificial Community		
	2020	2021		
Crop	FBO	FB	Oat	FBO
Sowing density	36 seeds m^-^² faba bean 136 seeds m^-^² oat	54 seeds m^-^²	408 seeds m^-^²	36 seeds m^-^² faba bean 136 seeds m^-^² oat
Sowing date	2020–03-31	2021–03-31		
Harvest date	2020–07-23	2021–08-16		
Preceding crop	winter rye (2019)	soybean		
weed control	none	selective		
Crop emergence	2020–04-20	2021–04-27		
Plant height				
1^st^ sampling date	2020–05-14	2021–05-14		
2^nd^ sampling date	2020–06-08	2021–05-28		
3^rd^ sampling date		2021–06-08		
4^th^ sampling date		2021–06-30		
5^th^ sampling date		2021–07-15		
Biomass				
1^st^ sampling date	2020–06-09	2021–05-27		
2^nd^ sampling date		2021–06-07		
3^rd^ sampling date		2021–06-21		
4^th^ sampling date		2021–07-05		
5^th^ sampling date		2021–07-20		
Cover				
1^st^ sampling date	2020–05-12			
2^nd^ sampling date	2020–06-08			

### 2.2 Artificial community

#### 2.2.1 Experimental field sites

The field experiment for the artificial weed community was conducted at the experimental and research station Campus Klein Altendorf, located in vicinity of Bonn, Germany (50°37’ N, 6°59’ E). A mean annual temperature of 9.6°C and a mean annual precipitation of 625 mm characterize the local climatic conditions. The soil type prevalent at the location is Haplic Luvisol, which is derived from loess deposits. The homogeneous soil conditions ensured better control of the artificially created weed communities, which is why this site was chosen.

#### 2.2.2 Selection of target weed species

The selection of target weed species for the artificial communities was based on various criteria. Target species were common species in central Europe and abundant in the natural weed community at Wiesengut, ensuring their native status and adaptation to faba bean, oat, and intercrop cultivation. Further, the species of the artificial community represent different taxonomic families and ecological strategy types (Grime, 1977). Finally, sufficient availability of high-quality seeds was required, as some species are not available from seed traders.

Based on these criteria, the five following species were selected: *Chenopodium album* L. (abbreviated as CA), *Lamium purpureum* L. (LP), *Stellaria media* (L.) Vill. (SM), *Vicia hirsuta* (L.) Gray (VH) and *Viola arvensis* Murray (VA). Ecological traits of the respective species are listed in **Table 2**.

**Table 2:** Primary strategy (Grime, 1977) (CR, competitor/ruderal; R, ruderal); competitive index (Marshall et al., 2003) with higher values indicating lower competitiveness; value for invertebrates and for seed-eating birds (Marshall et al., 2003) with number of starts corresponding to importance; indicator values (Ellenberg et al., 2001) with L, light; T, temperature; K, continentality; F, soil moisture; R, reaction; N, nitrogen (all ranging from 1 to 9), and S, soil salinity, with 0 = intolerant to salinity; X, indifferent; NA, not available.

	Primary strategy	Competitive index	Value for invertebrates	Importance for seed-eating birds	Ellenberg indicator values						
					L	T	K	F	R	N	S
*Chenopodium album*	CR	25	***	***	X	X	X	4	X	7	0
*Lamium purpureum*	R	62.5	**	–	7	5	3	5	7	7	0
*Stellaria media*	R	25	***	***	6	X	X	X	7	8	0
*Vicia hirsuta*	R	NA	NA	NA	7	6	5	4	X	4	0
*Viola arvensis*	R	250	–	**	6	5	X	X	X	X	0

Insect criteria is based on the number of insect species associated with particular weeds (0–5 species = –; 6–10 = *; 11–25 = **; 26+ = ***). The importance of the plant genus for seed-feeding birds where *** = important for >8 bird species; ** = important for 3–8 species and * = 1 or 2 species; – = not important).

#### 2.2.3 Setup

The experiment was conducted in three different spring crops (faba bean (FB), oat, intercrop of faba bean and oat (FBO)). As in the experiment with natural weeds, faba bean variety was ‘Fanfare’ and the oat variety was ‘Max’. The crops were sown with a Hege machine in three separate blocks (i.e. the crops were not randomized across the experiment). Each block comprised 48 plots á 1.5 m width x 2.0 m length, with 6 rows of crops and 10 rows of weeds (respectively two weed rows in between two crop rows). The coulters of the sowing machine were set 2 cm above the soil surface so that the weed seeds were deposited in a strip of respectively 3 cm. As a certain minimum volume of seeds is required to ensure an even distribution of the seed over the distribution cone to the downpipes, the weed seed was enriched with 50 g of wheat grit. This method was based on a study by Wilson et al. (1995), who conducted a field experiment in which weed seed was mixed with grit to ensure even distribution of seeds. Directly after sowing, nets were used to cover the plots until germination to protect the experiment from birds.

Each of the three separate blocks comprised two experimental factors with four replicates. Factor 1 comprised six levels consisting of the five individual weed species sown as single species (“monocultures”), and in addition, as the sixth level, as an equiproportional substitutive mixture composed of all five species, with proportions based on density (each of the five species with a proportion of 0.2). Factor 2 varied weed density with three levels.). Each variant (sole weed species or mixture) was sown in a high and a low density (Swanton et al., 2015), and a negative control (no sown weeds) was added as well. The high-density variant targeted 300 weed individuals per m² while the low-density variant targeted 150 individuals per m², with the required amount of seeds determined following germination tests. Within each of the three experimental blocks the variants (combination of the two experimental factors) were completely randomized.

Crop and weed seedlings were counted on the central square meter of each plot and all excess seedlings, as well as seedlings of other non-target species germinated from natural soil storage, were removed by hand. During the vegetation period, plots were checked and cleared of non-target weed species once a week.

#### 2.2.4 Data acquisition

Plant density of crops and weeds was counted in each central square meter of a plot. Plant height and biomass of crop and weed plants were determined 5 times (date 1 – date 5) (**Table 1**). Biomass samples were taken outside the central square meter to minimize disturbance of the area in which final crop yield was sampled. Four crop plants (or 2 plants of FB) and 4 weed plants (or 2 per species in mixtures) were collected per plot at each time point. Fresh weight and dry weight of the plants were determined and upscaled to g m^-^² by multiplying mean weights per plant by plant density (**Supplementary Material A6**). On June 2^nd^, weed cover of each species and crop was visually estimated (Lotz et al., 1994; Vitta and Quintanilla, 1996).

On August 16^th^ the central square meter of each plot was harvested and the yield parameters including fresh biomass, dry biomass, ears or pods per m², grains per ear or pod and grains per m² were determined.

### 2.3 Data analysis

#### 2.3.1 Statistical analysis

To test whether the loss of crop biomass decreases with increasing evenness of a weed community, several linear regression models were evaluated. The independent variables weed evenness and weed biomass were analyzed with and without taking interactions into account while the different sampling dates were considered as random factor with the *lme–*function of the package ‘lme4’ (Bates et al., 2015). Also crop density was included into the model as co-variate, but no significant effects were found with any of the terms that included crop density. Similar analyses were performed for crop grain yield and weed evenness. In addition, regression analyses were conducted for all dates separately as a strategy to determine if significance effects at individual dates might be hidden behind overall non-significant results. To test whether the effects of weed biomass and weed evenness can be disentangled in our study, regression analyses were also conducted for weed biomass as function of weed evenness.

The setup of the experiment with artificial weed community allowed investigating the competitive effects of the different weed species on crop yield separately as well. Therefore, the mean yield value of the control was subtracted from the yield values of the different variants with the different weed species (**Supplementary Material A2**). The data was then analyzed applying a two-factorial ANOVA with weed species and weed density (high, low, control) as factors.

All statistical analyses were conducted with the open source program R Studio (23.12.1) (R Core Team, 2020).

#### 2.3.2 Weed diversity measures

The diversity of the natural communities and the artificial weed communities in Mix treatments were characterized through the Shannon diversity index *H’* (Equation 2) and Pielou’s evenness index (Equations 1). In the artificial communities these indices were computed on both weed density and weed biomass. As the biomass samples in the natural communities was only separated between the three most abundant species and remaining weeds, the diversity indices for that experiment were computed based on the species-specific weed cover.


(1) $$Evenness=\;\frac{H'}{H_{max}};\;H_{max}=ln\left(S\right)$$

(2) $$H^{\prime }=-\sum_{i=1}^{S}p_{i}\;ln\left(p_{i}\right);p_{i}=\frac{N_{i}}{N}$$

Where *S* is the overall number of species, *H_max_* is the maximum diversity, *N* is the number of individuals, *N_i_* is the number of individuals of species *i* and *p_i_* is the relative ratio of species *i* between 0 and 1.

#### 2.3.3 Mixing effects

Because we sowed weeds as individual species as well as in mixture, an alternative way to test the effect of weed diversity on crop yield is to analyze absolute mixture effects of the weed communities in comparison to the average of the individual weed species. In particular, if evenness of the weed community is related to crop yield, we would expect the effect of individual weed species (i.e. evenness of 0) on crops yield to be more detrimental, on average, than of a mixed community. To analyze mixing effects of the weeds on crop yield in the artificial weed community experiments, the average of the crop yield values of the five species-specific plots (single weed species) were subtracted from the yield value of the respective spatially closest plots with mixed weed communities. This means that the crop yield values of the single weed species plots were calculated with a respective proportion of 0.2, which corresponds to the proportion of the sowing in the mixed variants. Subsequently, the mean values of these yield-differences (absolute mixture effects) were calculated for each experiment (FB, oat, FBO) and ANOVAs were performed to test the absolute mixture effects against zero.

## 3 Results

The results of the regression analyses and mixing effects are presented below, while results of the analyses of the separate competitive effects of the different weed species are described in the **Supplementary Material** (**Supplementary Material A2**).

### 3.1 Evenness

**Figure 1:**
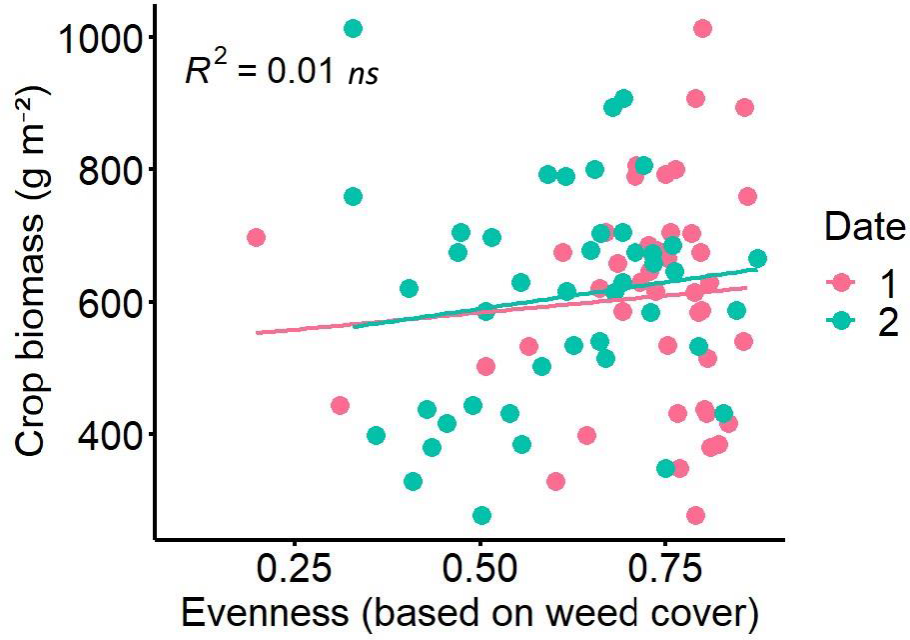
Crop biomass as function of evenness (based on weed cover) of natural weed community at Wiesengut in 2020 in a faba bean oats intercrop, with evenness measured on two different dates (2020–05-14, 2020–06-08) and *ns*, non-significant.

In the experiment with natural weed community weed evenness based on biomass ranged from 0.2 to 0.9 (**Figure 1**). Neither the regression analysis of crop biomass and weed evenness nor between grain yield and weed evenness showed any significant effects. Only a very slightly significant, positive relationship was found for faba bean grains (separated from the intercrop) with a p-value of 0.053 and R² of 0.086 but only with the evenness recorded on date 2. No significant relationships occurred for total grain yield of the intercrop of oat and faba bean and evenness at any date.

**Figure 2:**
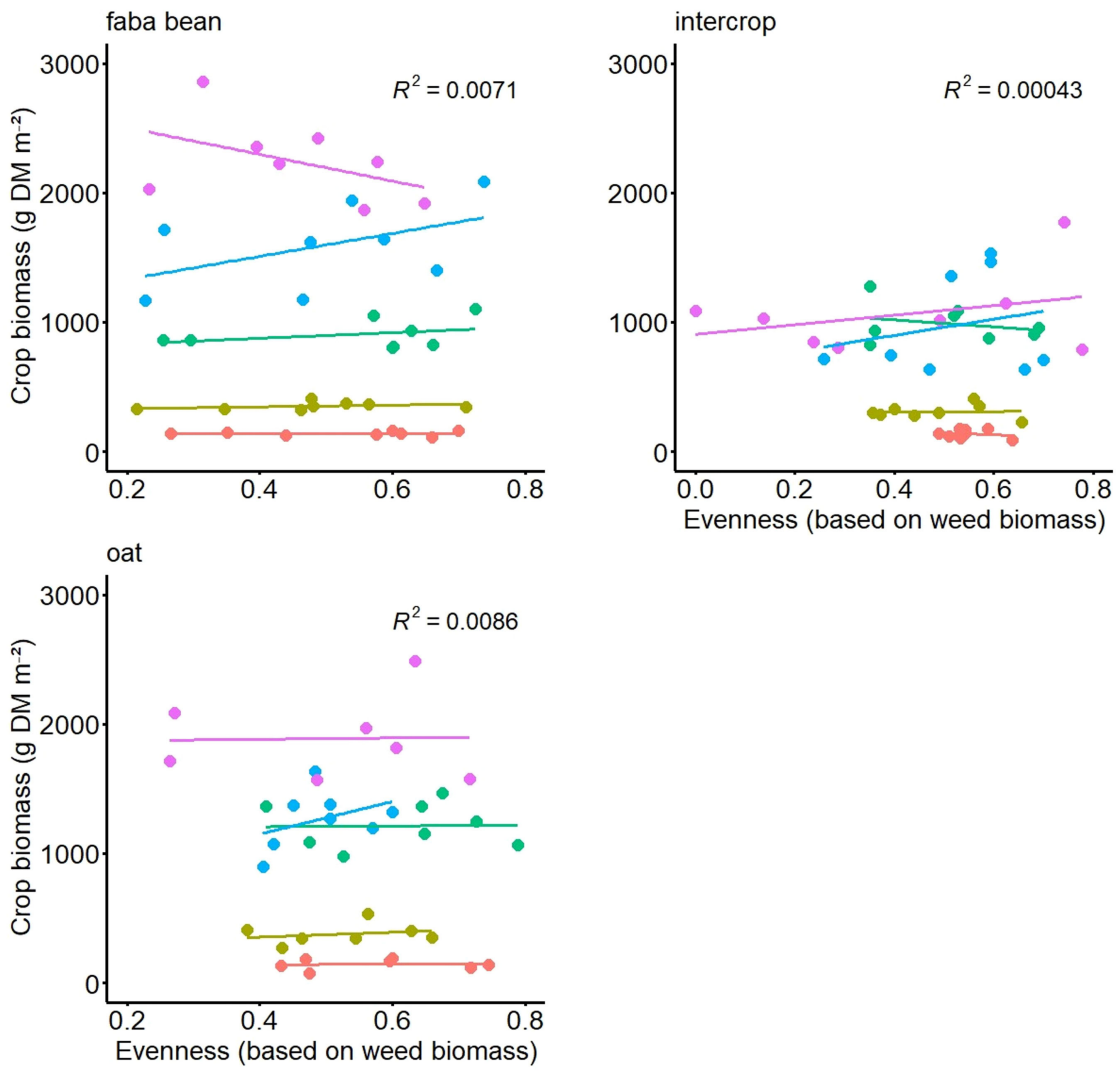
Crop biomass (dry matter, DM, in g per m^2^) as a function of evenness (based on weed biomass) of the artificial weed mixture at the five different sampling dates in three different crops, namely faba bean the faba bean oats intercrop and oats.

In the experiment with artificial weed communities, the regression analyses did not show any significant relationships between crop biomass (or grain yield) and weed evenness either. For none of the terms in the over-all model, i.e. when date was a random factor, the calculation of the linear regressions was significant in any of the three field trials (FB, oat, FBO). Although in individual cases, significant correlations were found for the relationship between crop biomass and both variables of weed evenness and weed biomass, these were not consistent. E.g. in FB this relationship was negative with a p-value of 0.02, but only for date 5 and in FBO it was positive in the model with date as random factor with a p-value 0.09 but was not consistent in the models of the respective dates. Weed evenness ranged from 0.2 to 0.7 in FB, 0 to 0.8 in FBO and 0.3 to 0.9 in oat (**Figure 2**). For the relationship between crop grain yield and weed evenness, no significant effects were found.

Regression analyses were also calculated with weed evenness based on weed density. However, no significant effects were detected with this analysis either. Regression analysis with weed biomass and weed evenness showed no significant correlations for either the natural or the artificial weed communities.

### 3.2 Mixing effects

**Figure 3:**
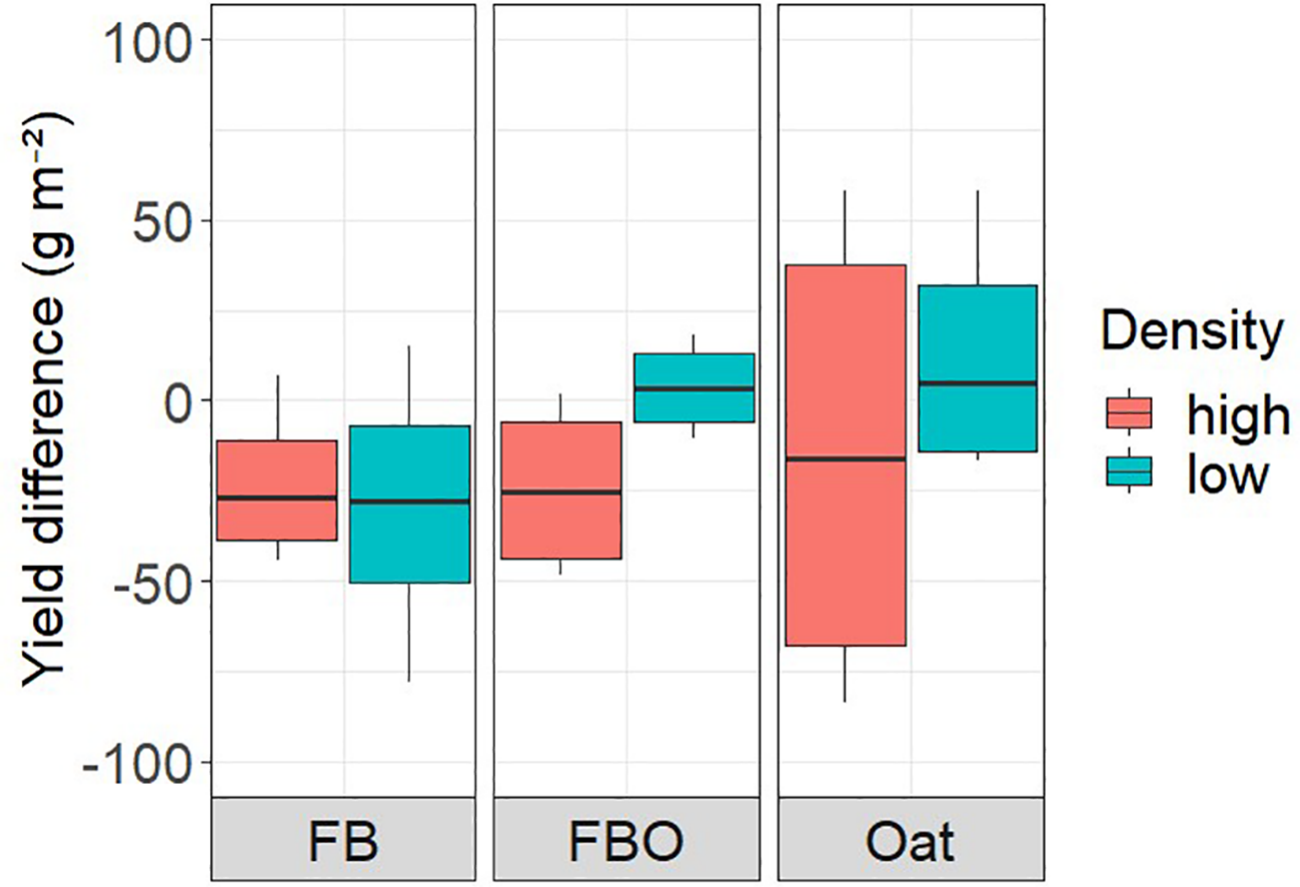
Difference between yield in weed mixture and yield in separately sown single weed species (CA, LP, SM, VA, VH) in high and low density variants the three crops (faba bean sole crop (FB), the intercrop of faba bean and oats (FBO), and the oats sole crop).

We did not detect any significant absolute mixture effects on crop yield when comparing mixed weed communities with the average of the single weed species (**Figure 3**). The absolute mixture effects were also not consistent across experiments and densities. In the faba bean crop, the average yield was lower in treatments with weed mixtures than with relative proportion of the single sown weeds in both density variants. In FBO and oat the yields of the species-specific variants in low density were just above those of the mix variants, while those in high density also showed a tendency for lower yields.

## 4 Discussion

Recent research in the field of integrated weed management suggests to support more diverse weed communities. Most prominent in this context might be the results of Adeux et al. (2019) who found in a comprehensive study that crop yield losses are mitigated through weed diversity. These findings are promising as they unite both objectives of high crop productivity on the one hand and biodiversity conservation on the other. Furthermore, the results indicate that the possibility of limited or even no intervention in a field depends on the present diversity and considering more precisely, on high weed evenness of the weed community.

The aim of this study was to investigate the relationship between crop biomass production and weed evenness and to test whether a positive relationship between both variables can be shown, as previously reported by some studies (Cierjacks et al., 2016; Adeux et al., 2019). However, neither the results of the experiments with natural nor those with artificial weed communities validated a higher crop biomass production when weed evenness was high. Since no crop yield losses occurred in any of the weed communities in our study, it was also not possible for mitigation of yield losses to occur, either due to evenness of the weed community or any other reasons. This raises the question why the various weed communities did not cause any yield losses. One reason might have been the low biomass production of the weeds in relation to the total biomass (crops and weeds). Weed biomass as a proportion of total biomass ranged between 1 and 27% at CKA (median of 4% across all values) and between 2 and 36% at WG (median of 8%). In comparison, in a study by Hyvönen and Salonen (2005) on weeds in different cereal cropping systems, values ranged between 4 and 11%, and a similar range (4 to 15%) was observed in an intercropping study by Corre-Hellou et al. (2011). In a further study (Szumigalski and van Acker, 2005) the range was larger (1 to 88%), with a median of 14.5%. While these comparisons show that our values are broadly comparable with values found in other investigations, the weed biomass found in our study might still not have been high enough to cause any yield losses.

Another possible explanation could be that other influencing factors may have superimposed or compensated the occurrence of competitive effects, e. g., the effects of extreme weather conditions, but as we argue below, this explanation is unlikely to be valid.

In 2021, the amount of precipitation was relatively high with 487 mm from January to July (long-term average: 345 mm) (**Supplementary Material A1**), which might have reduced competition between weeds and crops for water resources and enabled high crop biomass production although weeds were present (Kaur et al., 2018). In contrast to 2021, in 2020 there was a relatively low amount of precipitation with 354 mm (long-term average: 455 mm), so water was presumably a limiting growth factor (Iqbal and Wright, 1998). Nevertheless, no competitive effects between crops and weeds occurred in 2020, as both might have suffered from drought. Whether competitive effects actually occur in the vegetation does not only depend on the availability or deficiency of a resource but also on the capability of plants to use it (Patterson, 1995). If neither the crop plants nor the weed plants are able to absorb the resources in sufficient quantities, the coexistence is not dominated by one of the two. However, as our study was performed in two years with contrasting weather conditions, we think that this is unlikely to be the decisive reason for the lack of a significant relationship between crop biomass production and weed evenness.

Higher crop biomass productivity with lower weed evenness, i.e. an effect opposite to the one which would be expected due to niche complementarity, may also occur under favorable conditions with a sufficient supply of potentially growth-limiting resources as water (see above), nutrients and light (Craine and Dybzinski, 2013). Under these conditions, crop plants can build high biomass although weeds are present as competition for resources is weak. Independently of the lacking competitive effects between the crops and the weed community, different weed species within that community might still compete for resources. If conditions are favorable for crops, weed species that occupy a similar niche to the crop plant might find favorable conditions as well (Neve et al., 2009; Borgy et al., 2012; Storkey and Neve, 2018). This may then result in a weed community dominated by these adapted species and decrease the evenness of the weed community (Blackshaw and Brandt, 2008; Kordbacheh et al., 2023) while crop biomass remains high. This hypothesis is partly validated by the results of our field experiment in 2021 as the accompanying weed community of faba bean was highly dominated by *Chenopodium album* a species adapted to spring sown crops (Aper et al., 2012; Weber et al., 2017; Bajwa et al., 2019; Eslami and Ward, 2021); indeed, *C. album* produced 80% of the average weed biomass of the entire weed community.

Competitiveness not only depends on weed density, height and biomass but also on when these are established in relation to crop height and biomass (Valizadeh and Mirshekari, 2011). In a study of Boström et al. (2003) in which weed species were ranked according their association with the extent of yield loss, *Chenopodium album* and *Viola arvensis* did not, as assumed, emerge as important for predicting yield loss despite being abundant in the experiments. They explain this phenomenon with the fact that small-seeded species, like *C. album*, may take longer before they begin to interfere with the crop and, in the case of drought later in the spring, may already have ceased growing before any crop interference. However, in our experimental year 2021 there was no drought in spring so that this is unlikely to be the explanation in this case. However, crops can gain a decisive growth advantage at an early stage, with the potential to suppress the competition effects of the weeds. With such an advantage of the crop plants, the effect of high or low weed evenness might be of secondary importance for competitiveness on crop production. A comparison of the average height of crops and weeds in the artificial community experiment showed a large difference even at the first sampling date in all crops (14 cm vs. 2 cm in FBO; 9 cm vs. 2 cm in FB; 18 cm vs. 2 cm in oat). In this case weed evenness might indeed have been of secondary importance regarding the competitive ability of the weed community.

For the artificial weed community, the question arises if the target weed species generally hold too little competitive power for mitigation to occur in crop yield losses through weed diversity. However, the competitive indices defined by Marshall et al. (2003) for *Chenopodium album* and *Stellaria media* were exceeded in our experiment. Furthermore, *Chenopodium album* is listed as one of the ten most widely distributed and problematic weed species for several crops (Bajwa et al., 2019) which is why competitive effects would have generally been expected in our case as well. The lack of competitive effects, despite the weeds exceeding the published thresholds,, supports the assumption that competition effects were overlaid or compensated by other effects (Ingle et al., 1997; Bowerman and Whytock, 2000). Competition and compensation effects are strongly context dependent. We see a large gap in research on weed damage thresholds at a community level instead of at species level to predict in which weed community a certain weed species is detrimental and in which community this species is restricted in its competitive power and can be retained without crop yield losses. However, these effects are complex (Esposito et al., 2023) and it remains questionable whether this research gap can be closed satisfactorily at all. Just as essential ecological effects explain the emergence of competitive effects between crop yield and weeds, they explain the absence of these effects with high diversity of the weed community through niche complementarity, but they also explain the absence of these effects independent of a high diversity as found in this study.

Another hypothetical argument for our results may be that the variability of evenness found was just too low to show a positive relationship between crop biomass production and weed evenness. However, this was not the case. While in the study of Adeux et al. (2019), weed evenness varied from 0 to 1 with a mean value of 0.6, in our study evenness ranged from 0.2 to 0.9 in the natural weed community, from 0.2 to 0.7 in FB, from 0 to 0.8 in FBO and from 0.3 to 0.9 in oats; thus, in all cases, evenness varied substantially.

A limitation of our results might have been the methodological differences in the calculation of evenness between both field experiments, i.e. the natural and the artificial weed communities. As for the natural weed community no species-specific biomass values were available (except for the three most abundant species), weed evenness was calculated by using weed cover instead. Although the cover might be a less precise estimate of species abundance than the biomass due to subjectivity in data acquisition (Andújar et al., 2010), Chiarucci et al. (1999) showed a highly significant positive correlation between evenness values based on weed biomass and evenness values based on weed cover. Evenness based on weed cover was also used in further research, e.g. in a study by Chamorro et al. (2016) on organic farming effects on biodiversity in Northeast Spain. Furthermore, a regression analysis between estimated weed cover and weed biomass of the three most abundant weed species in the natural weed community at Wiesengut showed significantly positive relationships between both variables (**Supplementary Table A7**). This is similar to the results found by Andújar et al. (2010), where estimated weed cover and weed biomass were positively correlated. Despite these correlations, our results based on cover-based evenness need to be interpretated with caution.

In summary, we have investigated two environments with different soil properties in two years with contrasting weather conditions and with different management practices (organic, conventional) while variability of evenness was similar to the one found in the reference study (Adeux et al., 2019). In the different locations, we examined natural and artificial weed communities which included species with a high competitive potential, such as Chenopodium album, and in which the number of individuals at least partially exceeded the competitive indices described by Marshall et al. (2003). Despite this variety of conditions, we did not find any significant relationships between crop biomass production and weed evenness or, in fact, any detrimental effect of the weeds on the crop plants. We conclude that, while a diverse weed community might indeed have the potential to strongly reduce yield losses (Navas, 2012; Esposito et al., 2023), there are numerous conditions and compensatory effects under which this may not be observed. Possibly, the absence of competitive effects of weeds on crop biomass production is more likely when weed diversity is high. However, currently it is unclear under which (environmental) conditions this effect reliably occurs; it therefore may become of less relevance for the weed management at an individual farm. Furthermore, for farmers it is crucial whether the (potential) positive relationship between crop biomass production and weed evenness is also reflected in crop grain yield. However, neither such a positive relationship between crop grain yield and weed evenness could be shown in our study, nor was it reported in the study of Adeux et al. (2019).

In terms of methodology, we chose two complementary approaches, namely monitoring effects in naturally occurring weed communities across a heterogeneous field on the one hand and sowing single weed species and a defined artificial community of carefully selected, locally typical weed species, on the other. A further potential method would be to manipulate real weed communities to make them more even, by removing the dominant species; this has the advantage of maintaining species and genotypes adapted to site and management, and, while possibly labor intensive, is a possible avenue to explore the relationships between crop productivity and weed diversity in future research.

Although we investigated crop mixtures (faba bean plus oats) along with their respective monocultures in our study, the design of our experiment does unfortunately not allow us to make a direct comparison between the crop mixture and its components. In a way, our investigation is complementary to a study conducted in Switzerland and Spain by Stefan et al. (2021) where various crop mixtures were compared to their respective component sole crops with regard to their effects on weed communities, but where the composition of weed communities was not experimentally manipulated. There, intercropping was shown to reduce weed biomass and diversity in one country but not in the other. If both experimental approaches are combined to independently vary the diversity of both the weed community and the crops, it is currently difficult to predict outcomes of this complex and dynamic interplay between multiple partners. Using crop-weed models could help to form hypotheses in this case before embarking on empirical studies in the field.

In this study we showed that there are also weed communities of relatively low diversity, which do not have detrimental effects on crop productivity. With the calculation of the mixing effects of the weeds, we showed that even weeds in ‘monoculture’ did not cause significant yield losses compared to the weed community. This might indicate that at least in some contexts, non-intervention is not only possible with highly diverse weed communities but also with lower diversity and evenness. This is of particular importance since even low-diversity weed communities may make significant contributions to ecosystem functioning. For soil erosion control it may be of greater importance whether there is a sufficient soil cover than the occurrence of many different species or a high community evenness (Gyssels et al., 2005; Panagos et al., 2015; Lenka et al., 2017). Also, as resource for the associated biodiversity, mass flowering or a sufficient biomass of certain weed species can be beneficial or even decisive for the population development of associated species (Holzschuh et al., 2013), e.g. many phytophagous insects feed on just one or two plant families (Ward and Spalding, 1993). Therefore, the aim of weed management should be to preserve not only diverse communities, but in fact all non-competitive weed communities in the field as they are likely to contribute to ecosystem functions.

## 5 Conclusions

This study was based on the hypothesis that an in-field trade-off reduction between crop productivity and biodiversity conservation can be achieved by managing weeds towards a diverse community, which is supported by current literature in weed research. The results of this study do not underpin these findings, possibly due to low weed pressure and the absence of competitive effects, but rather indicate that also little diverse weed communities may be maintained without suffering yield losses in some cases. While the results based on weed cover as in the natural weed community need to be interpreted with caution, we consider our findings important as not only diverse weed communities contribute to ecosystem functions, but also higher abundances of few individual species, especially when considering not only weed diversity but also the associated diversity, which uses specific weed species as main food source or shelter and depend on minimum abundances of these species.

## Data availability statement

The raw data supporting the conclusions of this article will be made available by the authors, without undue reservation.

## Author contributions

MZ: Conceptualization, Data curation, Formal analysis, Investigation, Methodology, Software, Visualization, Writing – original draft, Writing – review & editing. TD: Conceptualization, Data curation, Funding acquisition, Project administration, Supervision, Validation, Writing – review & editing.
