# Supplementary material for: Does weed diversity mitigate yield losses?
Source: Front Plant Sci. 2024 Jul 12;15:1395393. doi: 10.3389/fpls.2024.1395393 (PMC11272534; doi:10.3389/fpls.2024.1395393)
Supplement: Supplementary file 1 [file DataSheet_1.docx]

**Appendices**

# Results

## Precipitation

The long-term average precipitation was calculated with data of the last 30 years for Wiesengut and with the last 60 years for Campus Klein Altendorf.

a)

b)

**Figure A1**: Average monthly and long-term average precipitation in 2020 at Wiesengut (a) and in 2021 at Campus Klein Altendorf (b)

## Species-specific yield effects

CA at high density (CA high) caused significantly lower yields than LP at high density and VA at low density (**Fig. A2a**). The yield in CA high was lower than all other treatments as well, but no significances were found. The yield in CA high was significantly lower than in the control about 27.7%. In order to explain the significant effects in FB, linear regression analyses between the yield differences and several weed parameters were calculated. The results showed high significant negative correlations between yield and biomass (p-value = 0.00006; df = 46; R² = 0.3) and yield and weed height (p-value = 0.0001; df = 46; R² = 0.28), whereas weed density and weed cover did not show any significances.

In FBO and oat, no treatment caused significant yield losses compared to the yield of the control. Differences between the treatments were not significant as well.


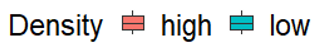


a)


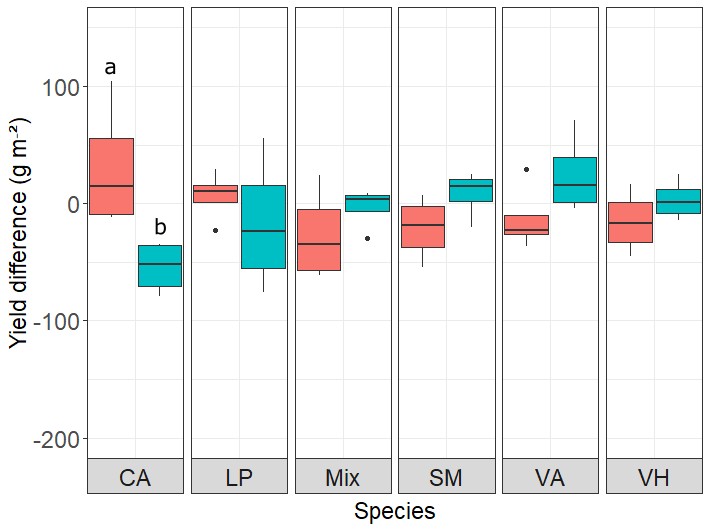

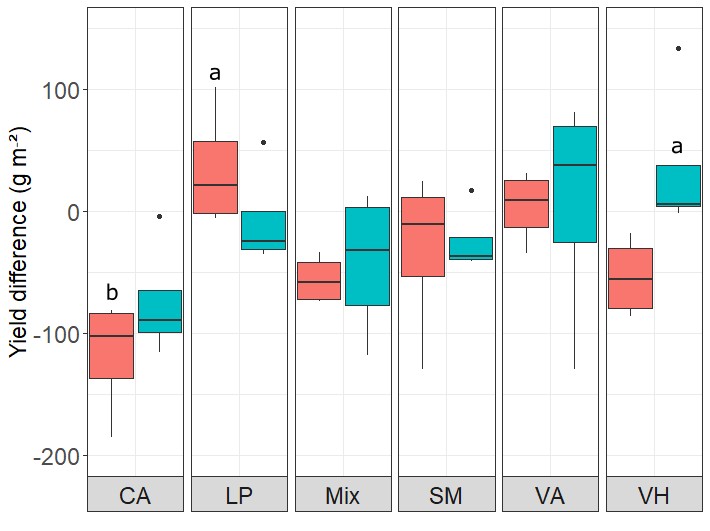

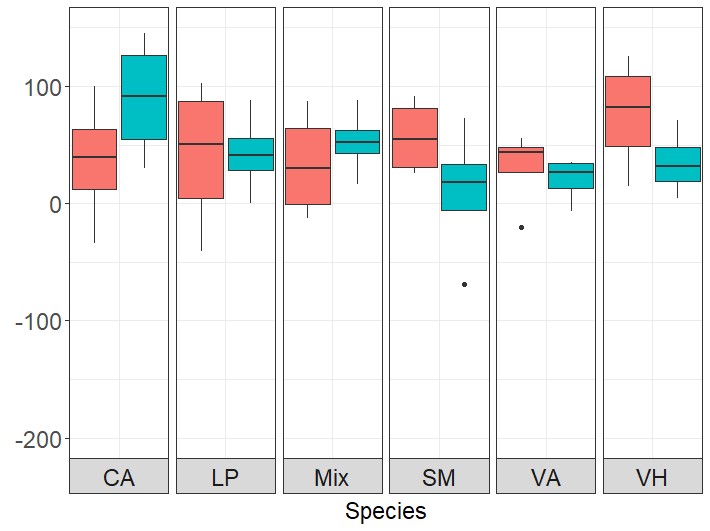


b)

c)

**Figure A2**: Differences in Yield between the different species in high (red) and low (green) density in FB (a), oat (b) and FBO (c). Letters indicate statistical differences between treatmens according to Tukey HSD test. Significance levels: 0 ‘***’ 0.001 ’**’ 0.01 ’*’ 0.05 ’.’ 0.1 ’’ 1.

## Species frequency of natural weed community at Wiesengut

**Figure A3**: Relative species frequency (n=44) of the natural weed community at Wiesengut in %.

## Soil properties at experimental sites

**Table A4**: Chemical soil properties at Wiesengut 2020 and Campus Klein Altendorf 2021.

|  | Wiesengut | Campus Klein Altendorf | | |
| --- | --- | --- | --- | --- |
|  | Intercrop | Faba bean | Oat | Intercrop |
| P (mg/100g soil) | 10.8 | 2.3 | 2.7 | 2.2 |
| K (mg/100g soil) | 7.9 | 53.9 | 15.9 | 34.7 |
| C/N ratio | 9.3 | 9.6 | 9.6 | 9.3 |
| pH (CaCl_2_) | 5.9 | 6.9 | 6.9 | 6.9 |

## Weed biomass as function of weed evenness

**
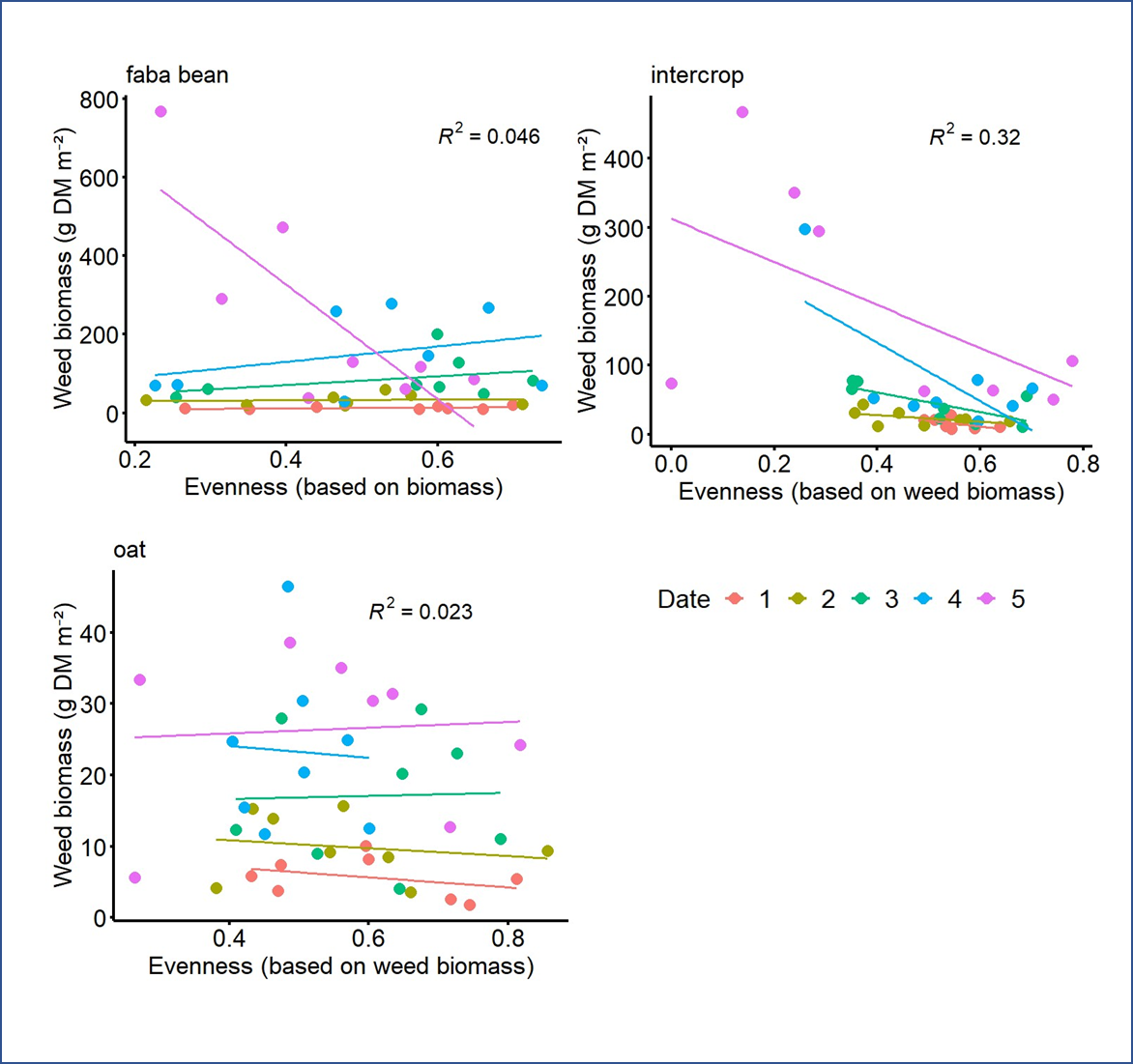
**

**Figure A5.1**: Weed biomass as functions of weed evenness (based on biomass) at Campus Klein Altendorf in the different crops and to the five different dates (note the different ranges of the axes).


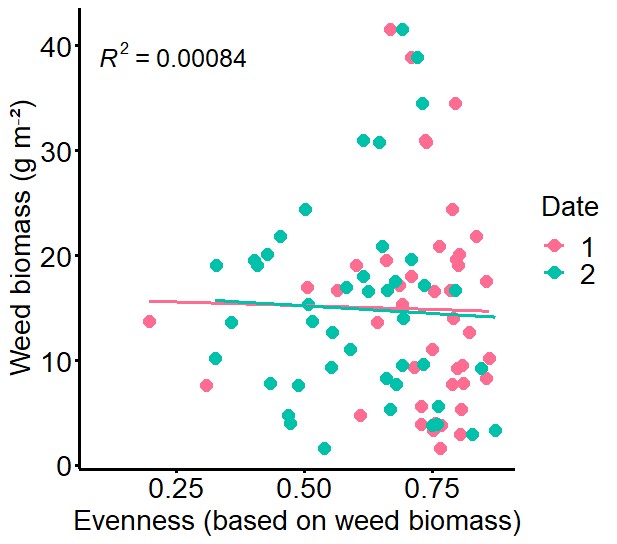


**Figure A5.2** Weed biomass as functions of weed evenness (based on biomass) at Wiesengut to the two different dates.

## Biomass of crops and weeds


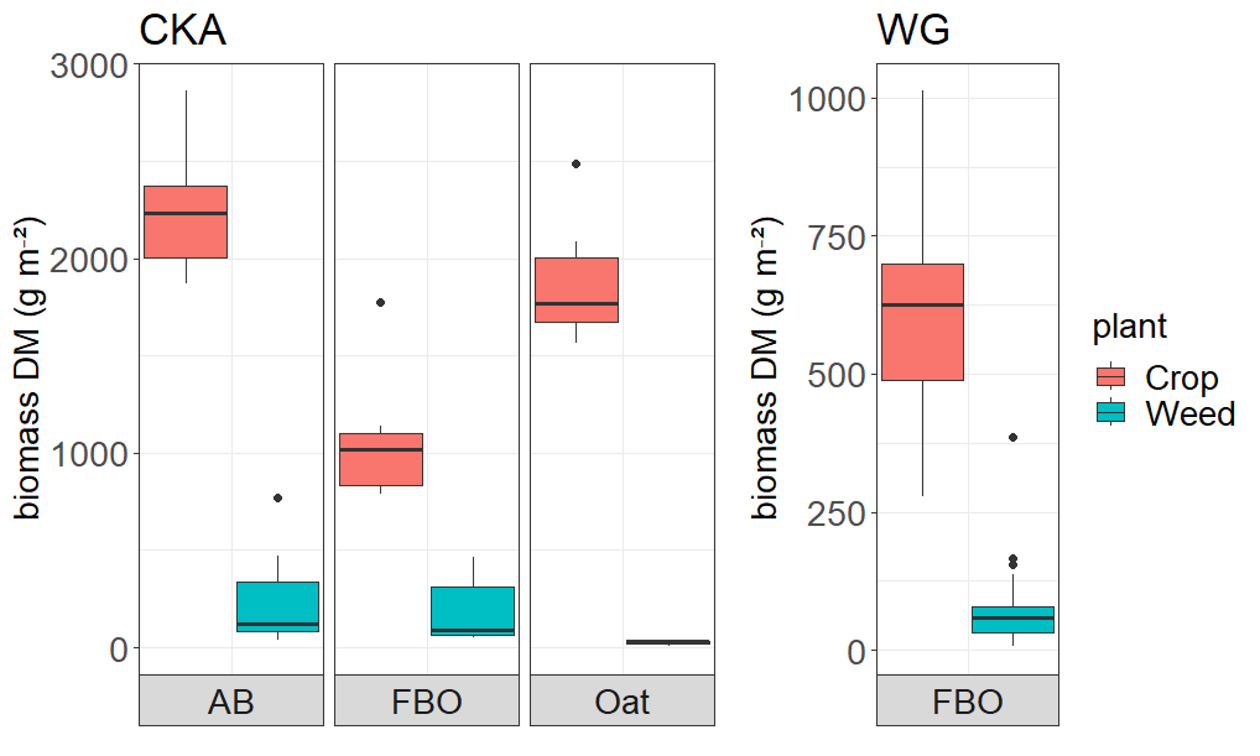


**Figure A6**: Biomass (DM) of crops and weeds (on sampling date 5) at Campus Klein Altendorf (CKA) in the different crops and at Wiesengut (WG) in intercrop.

## Relationship between weed cover and weed biomass

**Table A7**: Results of the regression analysis in the natural weed community at Wiesengut. Significance levels: ***: <0.001’. Estimate refers to the value of the slope for the regression of biomass against cover. Cover1 and Cover2 in the model refer to the percentage cover at date 1 and 2, respectively.

| Date | Model | Estimate | P-value | Significance |
| --- | --- | --- | --- | --- |
| Date 1 | Biomass ~ Cover1 + species | 1.142 | 2.73E-08 | *** |
| Date 2 | Biomass ~ Cover2 + species | 0.448 | 6.58E-09 | *** |
